# Barriers and facilitators to the integration of mental health services into primary healthcare: a qualitative study among Ugandan primary care providers using the COM-B framework

**DOI:** 10.1186/s12913-018-3684-7

**Published:** 2018-11-26

**Authors:** Edith K. Wakida, Celestino Obua, Godfrey Z. Rukundo, Samuel Maling, Zohray M. Talib, Elialilia S. Okello

**Affiliations:** 1 Department of Psychiatry, Mbarara University of Science and Technology, P. O. Box 1410, Mbarara, Uganda; 2 Department of Pharmacology and Therapeutics and Vice Chancellor, Mbarara University of Science and Technology, Mbarara, Uganda; 3 Department of Medical Education, California University of Science and Medicine, San Bernadino, California USA; 4 Mbarara University of Science & Technology, Mbarara, Uganda; 5 Department of Psychiatry, Makerere University College of Health Sciences, Kampala, Uganda

**Keywords:** Integration of mental health services, Primary care providers, Uganda clinical guidelines, Capability, Opportunity and motivation

## Abstract

**Background:**

Uptake of clinical guideline recommendations into routine practice requires changes in attitudes and behaviors of the health care providers. The World Health Organization (WHO) has heavily invested in public health and health promotion globally by developing policy recommendations to guide clinical practice; however, clinical guidelines are often not applied. The success of the implementation of any guidelines depends on consideration of existing barriers and adequately addressing them. Therefore, exploring the context specific barriers and facilitators affecting the primary care providers (PCPs) in Mbarara district, Uganda may provide a practical way of addressing the identified barriers thus influence the PCPs action towards integration of mental healthcare services into PHC.

**Methods:**

We adopted a theoretical model of behavior change; Capability, Opportunity and Motivation developed to understand behavior (COM-B). This was a cross-sectional study which involved using a semi-structured qualitative interview guide to conduct in-depth interviews with PCP’s (clinical officers, nurses and midwives).

**Results:**

*Capability* - inadequacy in knowledge about mental disorders; more comfortable managing patients with a mental problem diagnosis than making a new one; knowledge about mental health was gained during pre-service training; no senior cadre to consultations in mental health; and burdensome to consult the Uganda Clinical Guidelines (UCG). *Opportunity* - limited supply of hard copies of the UCG; guidelines not practical for local setting; did not regularly deal with clients having mental illness to foster routine usage of the UCG; no sensitization about the UCG to the intended users; and no cues at the health centers to remind the PCPs to use UCG. *Motivation* - did not feel self-reliant; not seen the UCG at their health facilities; lack of trained mental health specialists; conflicting priorities; and no regulatory measures to encourage screening for mental health.

**Conclusions:**

Efforts to achieve successful integration of mental health services into PHC need to fit in the context of the implementers; thus the need to adapt the UCG into local context, have cues to enforce implementation, and optimize the available expertize (mental healthcare providers) in the process.

**Electronic supplementary material:**

The online version of this article (10.1186/s12913-018-3684-7) contains supplementary material, which is available to authorized users.

## Background

Uptake of clinical guideline recommendations into routine practice requires changes in attitudes and behaviors of the health care providers as well as structural changes in their work environment [1, 2]. Evidence suggests that in order to promote adherence to guideline recommendations, there is need for structural adaptation to specific settings and target populations at different levels [3, 4].

The World Health Organization (WHO) has heavily invested in public health and health promotion globally by developing policy recommendations to guide clinical practice [5], and one such investment is promotion of mental health services into primary healthcare (PHC). Through the Alma-Ata declaration [6] PHC was adapted as the preferred method for providing a comprehensive, universal, equitable and affordable healthcare that could reduce stigma, improve access to care, reduce chronicity of mental illness and improve social integration [7–9]. In the Alma-Ata declaration, it was recommended that countries transform their mental health services to: i) promote self-care, ii) build informal community care services, iii) build community mental health services, iv) develop mental health services in general hospitals, and v) limit reliance on psychiatric hospitals [10]. In addition, WHO issued recommendations to guide the integration process at PHC level [9] because it was seen as the most viable way of closing the treatment gap and ensuring that people get the mental healthcare services they need [11]. Integration of mental health services into PHC has been embraced by various countries and in different forms [12–15] including a) training primary care providers (PCPs) in identifying mental health problems; b) PCPs assessing for mental illnesses during medical standard of care; c) PCPs/Community Health Workers (CHWs) and health care managers working together to address mental health related illnesses; and d) availing psychotropic medications to PHC centers [16]. Despite all these efforts, PHC for mental health has not been realized in most countries of the world; it is estimated that the treatment gap is widest among people with severe mental disorders in least resourced countries [11, 17–19].

In Uganda, mental health problems are recognized as a public health challenge contributing 13% to the national disease burden [20–22], and as such the Ministry of Health made health reforms to adopt the WHO recommendations of integrating mental health services into PHC [9, 11], these included: decentralizing the health delivery structures to the lowest level of care [11, 23]; formulation of the Uganda Minimum Health Care Package (UMHCP) with mental health as a key component [23, 24]; developing the Uganda Clinical Guidelines (UCG) on the management of common disorders [25, 26]; and training PCPs to identify and manage mental health problems, and refer complicated cases to higher levels of care [11]. However, there is still a gap in provision of mental health services at PHC inspite of all these reforms and the fact that research evidence suggests that mental healthcare can be delivered effectively in PHC settings [11, 17, 27, 28].

Furthermore, literature shows that clinical guidelines are often not applied and that the success of their implementation depends on consideration of existing barriers and adequately addressing them [29]. Interventions directed towards specific barriers are more effective than those that are not, thus the need for a planned introduction of tailored guidelines that are culturally sensitive to the targeted population [4, 30]. This study was developed following our earlier study about the need to conduct context specific studies to identify the barriers and facilitators the PCPs face in integrating mental health services into PHC; Wakida EK, Talib ZM, Akena D, Okello ES, Kinengyere A, Obua C. Barriers and facilitators to the integration of mental health services into primary health care: A systematic review [Forthcoming].

Using a case study approach [31, 32] and the Capability, Opportunity and Motivation framework for understanding behavior [33] the aim of this study was to explore the context specific factors affecting the ability of PCPs in rural Mbarara district to integrate mental health services into PHC. Understanding these factors may provide a practical way of addressing the identified barriers thus influence the PCPs action towards integration of mental healthcare services into PHC. In addition, conclusions from the findings may make it possible to design a relevant intervention tailored to the context of the PCPs in order to promote better implementation of the policy option. In the Ugandan healthcare system, PCPs, including medical officers, clinical officers, nursing officers and midwives are directly involved in assessing and managing mental health problems.

### Theoretical framework

**Fig. 1:**
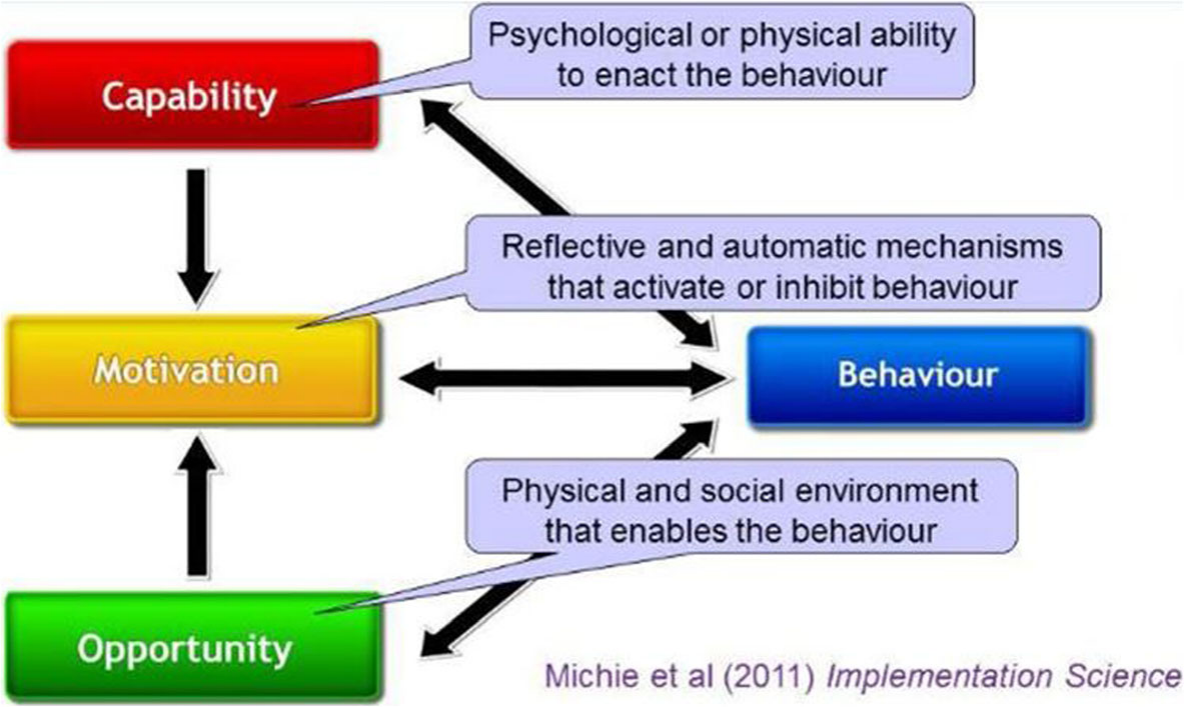
COM-B Framework for understanding behavior

In this study we adopted a theoretical model of behavior change; Capability, Opportunity and Motivation developed to understand behavior (COM-B) [33–35]. The theory postulates that in order to change behavior, there should be an interaction between one or more of ‘capability’ to perform the behavior and/or ‘opportunity’ and ‘motivation’ to carry out the behavior (Fig. 1). According to the proponents of this theory [33], *Capability* is the individual’s psychological and physical capacity to engage in an activity (knowledge and skills); *Motivation* are brain processes that direct behavior, (goals, conscious decision-making, habitual processes, emotional responding, and analytical decision-making); while *Opportunity* are the factors (physical or social environment) that make the behavior possible or prompt it. In Fig. 1, motivation is influenced by both capability and opportunity, therefore enacting a behavior can alter capability, motivation, and opportunity. Emergent barriers and facilitators were identified using this framework.

## Methods

### Study design

This was a qualitative study that used thematic analysis to examine the barriers and facilitators to the integration of mental health services into PHC by PCPs in rural Mbarara district, Uganda. We used the a semi-structured interview guide to enable us understand the phenomena in its natural environment [32, 36]. The study was designed by EW in consultation with ESO, ZT, and CO; questions were developed based on the COM-B framework to enable us pick the emergent factors influencing the PCPs behavior towards the policy option. The interview guide was pilot tested at a HC that was not included in the study, and revisions made based on lessons learned.

### Study setting

The study was conducted in Mbarara district approximately 270 km (170 miles), by road, southwest of the capital city, Kampala. Mbarara is the administrative capital of southwestern Uganda and it boarders Ibanda and Kiruhura Districts to the north, Kiruhura and Isingiro Districts to the east, Isingiro and Ntungamo Districts to the south, and Sheema District to the west [37]. Demographically, Mbarara district lies between coordinates 00 36S, 30 36E and covers an area of 1846.4 km^2^ with a population of 472,625 of which 242,547 (51.3%) are females [37].

In Uganda provision of mental health services begins at health center III (sub-county level) and with subsequent referrals to HC IV (county level), district hospitals, regional referral hospitals and finally to the national referral hospital [26, 38]. Each health facility level (except HC III) is expected to have general doctors (medical officers), clinical officers (Diploma level Medical Assistants), nurses and midwives, and psychiatric nurses. The HC IIIs do not have general doctors but have all the other cadres of service providers. Mbarara district has 13 HC IIIs and four HC IV; provision of health services is spearheaded by the district health department responsible for curative and preventive healthcare [39]. All the HC, as the primary health facilities, that were included in this study are located in rural Mbarara district.

### Participant’s recruitment

Participants who took part in the study included clinical officers, nurses and midwives from six health centers (III and IV); doctors were not part of the study because the HCs either did not have general doctors in the establishment (all HC III), did not provide consent to participate or were not available at the time of the study (on official assignment off station). There are four HC IV in Mbarara district and each has only one Medical doctor.

The sampling frame for our study was 17 HCs (13 HC III and four HC IV). Each category (HC III and HC IV) was evaluated for inclusion based on whether they were a government facility, had PCPs’ who directly assessed patients, and not neighboring a similar health facility (including privately owned), or not located near a hospital. We randomized the facilities by strata (HC III and HC IV) to obtain an equal number of HCs per cluster.

While we proposed to consider age, gender, occupation and seniority/experience when selecting participants, we found on the ground that the health facilities had a smaller health workforce than we had anticipated (about five to eight per HC), thus we recruited all PCPs we found at the facilities and only interviewed those who provided signed consent. A total of 20 in-depth interviews were conducted, 12 participants were from HC IV and eight from HC III. In our view, 20 is sufficient to exhaustively generate information, and by the time we interviewed the last participant, we were eliciting similar responses with no new information being identified. In terms of gender we had more females (*n* = 18) than males (*n* = 2). There were no significant age differences between the study participants with ages ranging between 30 and 49 years at the time of the study (Table 1). In terms of position or health cadre’s levels, we found 10 nurses, four midwives, two psychiatric nurses, and four Clinical Officers in the health facilities. Views of all the participants were included in the analysis and contribute to the conclusions in our study.

**Table 1:** Summary of participants characteristics

Participant	Age	Gender	Health cadre	Level of education
P1	38	Female	Nursing officer	Diploma in Nursing and Midwifery
P2	41	Male	Clinical officer	Degree in Public Health
P3	31	Female	Enrolled nurse	Certificate in Nursing
P4	32	Female	Psychiatric nurse	Certificate in Mental Health Nursing
P5	32	Female	Nursing officer	Diploma in Nursing
P6	45	Female	Senior Nursing officer	Diploma in Nursing & Health service Management
P7	39	Female	Midwife	Certificate in Midwifery
P8	49	Female	Enrolled nurse	Certificate in Nursing
P9	32	Male	Clinical officer	Diploma in Clinical Medicine
P10	30	Female	Midwife	Certificate in Midwifery
P11	35	Female	Enrolled nurse	Certificate in Nursing
P12	49	Female	Nursing officer	Diploma in Nursing and Midwifery
P13	31	Female	Psychiatric nurse	Diploma in Mental Health Nursing
P14	38	Female	Senior Nursing officer	Diploma in Nursing
P15	32	Female	Midwife	Certificate in Midwifery
P16	32	Female	Clinical officer	Degree in Public Health
P17	38	Female	Clinical officer	Diploma in Clinical Medicine
P18	30	Female	Nursing officer	Diploma in Nursing
P19	47	Female	Midwife	Certificate in Midwifery
P20	30	Female	Enrolled nurse	Certificate in Nursing

### Procedure

In-depth interviews were conducted by the lead author (EW) and two trained research assistants (MN and CK) between November 2017 and April 2018. Each interview lasted approximately 60 min and was audio recorded. All interviews were conducted in English the national official language, and backed by field notes.

### Data collection and tools

A semi-structured interview guide was developed by EW in consultation with ESO, CO, and ZT; questions were developed based on the capability, opportunity and motivation framework for understanding behavior (Additional file 1). They focused on the use of existing Uganda Clinical Guidelines (UCG) when assessing for mental health problems. The UCG helps clinicians by presenting updated, practical, and useful information on the diagnosis and management of common conditions in Uganda. The interview guide was pilot tested at a health facility that was not included in the study.

### Data management and analysis

Data were transcribed verbatim by the research assistants, and checked by EW against the audio recordings for correctness of information before proceeding to the next set of interviews. Using the first set of transcripts, clarification was sought from ESO, CO, and ZT, to ensure that the questions were being asked and responded to in the correct way and would answer the research question. Data were thematically analyzed [40] with the help of a qualitative software Atlas.ti version 7 [41]. ESO and EW independently read through transcripts and developed codes in accordance with the COM-B domains capability, opportunity and motivation and the initial coding done by EW. The coding process was discussed with CO a senior researcher, and ZT a health policy expert. There was an iterative process during the coding to agree on which responses were either barriers or facilitators and which ones belonged to the capability, opportunity and motivation domains.

### Ethical considerations

The study was approved by the Gulu University Research Ethics committee (GUREC), and the Uganda National Council of Science and Technology (UNCST). Permission to conduct interviews in the Health Centers in Mbarara was obtained from the District Health Officer. All participants provided written informed consent was obtained before each in-depth interview. Privacy of the participants was ensured by not including identifiable information in addition to conducting the interviews in private space. We respected individual autonomy to participate in the study by not including those who declined to participate, all who consented to participate were informed about their freedom to withdraw from the study at any time; no participant withdrew from the study. All the audio recorded material and transcripts were kept by to the lead author (EW).

## Results

**Fig. 2:**
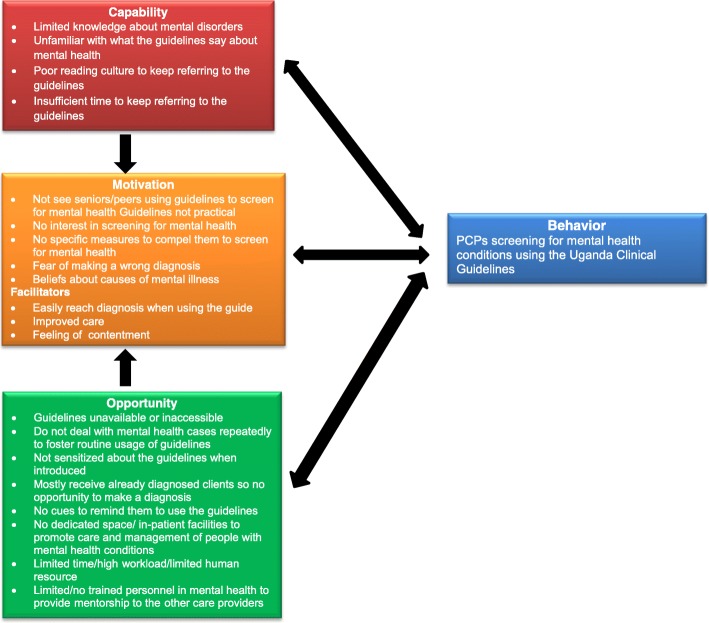
Emergent barriers and facilitators

We structured the identified barriers and facilitators (Fig. 2) around three domains of behavior change: i) Capability which is the individual’s psychological and physical capacity to engage in the activity concerned, ii) Opportunity - all the factors that lie outside the individual that make the behavior possible or prompt it, and iii) Motivation - all those brain processes that energize and direct behavior, not just goals and conscious decision-making. Although our participants across the HC comprised of 14 nurses with different levels of training and seniority (2 Senior Nursing Officers and 4 Nursing Officers, 4 Enrolled Nurses, 2 Psychiatric Nurses), 4 Midwives, and 4 Clinical Officers; the results presented in this section generally cut across the participants regardless of health cadre and facility level (HC III or IV). This could be explained by the fact that nearly all PCPs at different levels performed the same tasks of assessing patients and multitasking in addition to their other roles.

### Capability

The ability of PCPs to integrate mental health services into PHC was explored; majority of the participants expressed inadequacy in knowledge about the various mental disorders, causes and management. This was attributed to the fact that during their training, they studied purely to pass exams. Patients with mental illness are therefore treated as any other patients without paying attention to their mental condition.

I can make a diagnosis of mental illness, but because they are very many types, I may not be able to differentiate them if the signs are not clear…when we were in school (medical) we read to pass exams and then we come here and we are supposed to diagnose for mental illness; but that was not the focus so we just treat (mental health problems) like any other minor illness (Clinical Officer Health Facility (HF) 2)*.*

Participants seemed more comfortable managing patients with a diagnosis of mental health problems than making a new one. We found that the health centers received patients who had received a diagnosis directly from Mbarara Regional Referral Hospital and were coming for medicine refills; thus preferred to handle such cases.

As I told you, for us we do the basics then the rest refer. I think even the structures we have cannot allow us to handle those people from here completely. We don’t have isolation rooms for those people that’s why I say we cannot manage them unless they are treated from the other side and when they calm down, we can continue with their treatment (Nursing Officer HF 1)*.*

In addition, the knowledge about mental health that the participants had was gained during pre-service training; we found that since they started clinical practice, they had not received in-service training in the mental health area.

I can say that the knowledge I have on mental illness I got during my training. We do not have in-service training about mental health or the updates; at least if we are updated and we have that knowledge, we can handle (Nursing Officer HF 2)*.*

There was no senior cadre to seek consultations when not sure mental condition hence referrals of clients who should have otherwise been managed at the lower level. The participants indicated that they preferred management of patients already on treatment.

I do not think am very comfortable making a diagnosis of mental health problems…there is no colleague to ask if I find something confusing, so I refer to Mbarara Regional Referral Hospital because I know there are specialists there (Clinical Officer HF 1)*.*

When asked if they knew about the UCG and how helpful they could be when assessing for mental health problems, we found that the PCPs were aware about the UCG but found it burdensome to consult them unless they were totally in a fix as illustrated below by one participant.

To tell the truth it is not my culture to look through those guidelines (UCG) unless I am really cornered with a mother having signs of mental (Midwife HF 6)*.*

### Opportunity

We found that not all the PCPs had the opportunity to access the UCG because of the limited number of hard copies supplied to the respective health centers to be shared by all. This posed a challenge of knowing who last used the available copy and where it was kept in case they needed to refer to them.

When you get a client with signs of mental illness, you have to look for the guidelines (UCG), however, this process delays the patient or management of their condition; and it’s a challenge when you also have to look for the dose, calculate, and then think about the side effects of the drug (Midwife HF 3)*.*

The participants who had gained access to the UCG alluded to the fact that they were not practical for use in the local setting and needed to be summarized in a chart form to help them make the correct assessment of patients they suspect could be presenting with signs of mental illness. They noted that the UCG were very detailed and that the content about mental health was hidden and not quickly accessed when required.

Do you see how they have done for HIV care summarizing information on the charts and pinning everywhere…do the same for mental health. I don’t think I can leave a patient in front of me that I am looking for the guidelines (UCG) to read, they need a lot of time to search for information (Clinical Officer HF 6)*.*

The PCPs added that they did not repeatedly deal with clients having mental illness to foster routine usage of the UCG, thus found it bothersome to make reference each time the need arose. The health centers largely receive clients already diagnosed with mental health problems, and only need refills of their medication; there is limited opportunity for the PCPs to make a new diagnosis of mental health problems.

If we were seeing patients with mental illness on a daily basis with the same complaint, we would get used to the guidelines (UCG), but the patients (with mental health related problems) are not common…we mostly receive those with a diagnosis for refills so it is a challenge when we have to go and read the guidelines because we are not used to those patients (Midwife HF 4)*.*

Exacerbating the PCPs challenges of integrating mental health services into PHC is that there was neither sensitization about the UCG to the intended users nor provision of sufficient copies to promote self-sensitization.

We were just told that there are new guidelines to follow…they did not tell us how to use them and also did not give us personal copies. If at lease each one has a copy on their table, then maybe we can read them (Enrolled Nurse HF 1)*.*

There were no cues at the health centers to remind the PCPs to use UCG when assessing for mental health problems. The PCPs felt that mental health was not as important as other disease where a lot of attention and resources were directed. Some of the health centers had donor funded projects with a set agenda and necessary resources to achieve the aims; this was not known to some participants thus causing them to think that mental healthcare is neglected and therefore not important.

Nobody asks us about people with mental problems the way they follow up with other programs like HIV, TB, malaria and immunization…I think if the ministry wants us to comply with mental health, let them support it like the other programs (Nursing Officer HF 4)*.*

The other barrier related to opportunity was that the PCPs who were trained as mental healthcare providers and posted to the various health centers in rural Uganda were not necessarily practicing in their area of training. This caused the PCPs to feel deprived of time to do what they would be happy doing. The mental health integration policy requires all healthcare providers at PHC (lower levels of healthcare) to assess for mental health problems within routine practice. This in a way defeats the purpose of specialized training to practice at lower levels of healthcare where ones mental health should seamlessly be assed at PHC level.

I was posted here as a psychiatric nurse, but I don’t work as a psychiatric nurse, I rotate on all wards apart from maternity ward…I don’t have enough time to talk to these patients so that they know more about mental illness (Psychiatric nurse HF 3)*.*

The participants decried missed opportunity to provide mental health education to the patients they believed were in the communities and who may neither be aware about their mental illness nor the possibility of having it attended to. The PCPs attributed it to the lack of protected time to practice in the area of training as well as facilitation for mental health outreach activities.

…patients are in the community but they don’t know about mental health concerns. They don’t know that even mentally ill patients can be given treatment and improve to become important. I need to be supported go via the community and talk to the people; but that gap isn’t there to go and talk to the people. Need to go to the churches and to different places and talk to the community (Psychiatric nurse HF 2)*.*

Community outreach programs such as immunization are supported by the Ministry of Health; when asked why they did not utilize that opportunity to integrate mental health education, the participants’ indicated that they would love to do it but because of limited time given and few personnel they are unable to include any other programs.

On the facilitative side, we found that some of the PCPs who used the UCG found them useful when screening for mental health problems. However, they alluded to challenges when it came to management of the patient after diagnosis.

The guidelines (UCG) are helpful because it is not something very hard to follow, you can reach the diagnosis easily… you face it when you do not have the drugs that you would have given the client and you opt for some simple drugs like diazepam instead of the real treatment (Nursing Officer HF 6)*.*

### Motivation

In this study, we found that some PCPs were not motivated to screen for mental illness using the UCG because they did not feel self-reliant. It could be attributed to limited knowledge and skills in both using the guidelines, and dealing with mental health problems.

I am not confident using the guidelines (UCG) because of the difficult terms in mental health... we were not taken through the guidelines to understand the terms and how to use them (Nursing Officer HF 5)*.*

Some PCPs had not even seen the UCG at their respective health facilities. This probably was because of either the limited supply of hard copies or lack of knowledge that they were supposed to use the guidelines when assessing patients.

…*personally, I have not seen those guidelines (UCG) in the room where I work from, I read them once when I was in Mbarara (Mbarara Regional Referral Hospital) like 3 years back (Enrolled Nurse HF 4).*

When asked if screening for mental health problems caused any emotions, the PCPs response was to the affirmative although they indicated that it could not deter them from doing their job as healthcare providers. Emotions were mainly expressed by the female participants who felt distressed especially when dealing with children or unaccompanied persons.

…I imagine like if this is my child in that condition, I feel like crying…I feel pity for the family. But again as a health worker of course we should not allow emotions to control us…I go ahead and see this client, I do not sit and cry with them…I try to counsel the patient until they are well (Nursing Officer HF 6)*.*

Lack of trained mental health specialists at the health centers is another barrier we identified to the integration of mental health services into PHC. Some PCPs did not feel motivated to uptake the policy option because they did not have mental health specialists for immediate consultation. As remedy to that barrier, they proposed continuous medical education, and refresher courses in mental health to help them perform effectively.

…we do not have a psychiatric nurse to inquire about the difficult terms in mental health that we do not understand; we need CMEs or refresher courses (Midwife HF 4)*.*

We found that integration of mental healthcare into routine care has been met with conflicting priorities thus limiting time for effective health education as expressed below.

I don’t health educate mental health on a daily basis because there are other diseases to health educate in like diabetes and hypertension. But after getting those people from the general patients, I put them aside and talk about mental illness because that is my specialty where I talk much (Psychiatric nurse HF 3)*.*

Integration of mental health services into PHC is a policy option in Uganda and most of the PCPs are aware that they are supposed to use the UCG when assessing for mental illness. However, there were no regulatory measures at the health facilities to encourage them screen for mental health problems.

There is nothing (measures) on ground to make us use the UCG. Maybe other units have but for us here we have not seen anything like that, not even in-service training in mental health. You are even the first person to come here in so many years on this mental health (Clinical Officer HF 6)*.*

On the facilitative side, the PCPs were cognizant of the fact that if they followed the UCG, there would be improved care of the patients.

I think it will improve the care of people with the mental illness….we may not miss out on the cause and the management as well as the dosing (Psychiatric nurse HF 3)*.*

In addition, some participants indicated that they would feel contented with the service offered if they followed the UCG.

...if I know I have done what I am supposed to do confidently and I do it for real, I feel very good because I will have given a good quality of service (Nursing Officer HF 6)*.*

When asked what kind of support was needed to enable them comfortably use the UCG when screening for mental health problems, one participant indicated that:

We need to be mentored on the assessment and management of people with mental illness so that it is easier for us when reading the guidelines (UCG) and managing or assessing for mental conditions (Nursing Officer HF 2)*.*

### Recommendations from participants

Notable recommendations from the study participants included the need to provide the PCPs with: a) copies of the UCG, b) summarized UCG for easier reference, c) in-service training for mental health, d) mental healthcare providers at each health center, and e) protected time for mental healthcare providers.

## Discussion

In this study, we were interested in understanding the behavior of PCPs towards integration of mental health services into PHC. Cognizant of the fact that behaviors occur within a context of other behaviors, we found it necessary to identify the specific factors that affect the PCPs implementation of the policy option [35]. Procedurally, we used the COM-B framework to develop the interview guide and structure the analysis. The advantage of doing this was that we understood the factors affecting the PCPs implementation of mental health integration into PHC, and identified the behavior *(PCPs following the UCG when assessing for mental health problems)* that needs to be addressed [33]. The COM-B framework has been used by various researchers, however, the closest study we found using the same approach was looking at barriers and facilitators to implementation of a web-based tool for diagnosis and monitoring of patients with depression [42].

Uganda is among the countries that undertook mental health reforms in conformity with a health policy philosophy which emphasizes decentralization of services to the lower administrative units. As a result, the UCG were developed with the aim to provide easy-to-use, practical and useful information on how to correctly diagnose and manage all common conditions [26] and avoid inappropriate variability in practice [43]. While clinical guidelines are systematically developed statements meant to assist practitioners in clinical decisions by providing cues to diagnosis and management of specific health conditions [1], we found in this study that most practitioners never found time to refer to the UCG. This was either due to lack of copies of the guidelines or conscious reluctance to refer to the guidelines [1].

### Barriers related to knowledge

Our study confirms a knowledge gap among the PCPs in relation to mental disorders, the UCG for management of common disorders, and what they say about mental disorders at PHC. This result aligns with several studies [44–59] that speak about barriers related to the PCPs knowledge and skills in integration of mental health services into PHC; there is lack of belief that they are capable of adequately performing the recommendation [60].

### Barriers related to accessibility of the clinical guidelines

Concerning accessibility to the UCG, there was limited access whether by shared or personal copy because of the limited supply to the health centers; this was compounded by lack of sensitization about the guidelines at the point of introduction. There is need for sensitization of the PCPs to the UCG communicating the rationale for introducing evidence, and implementation of the guidelines [61, 62]; involvement of active efforts to raise awareness and promote interest in the UCG; as well as proactive efforts to understand the needs of the user and follow through to achieve a change in behavior [63].

The PCPs suggested that they would like a simplified format of the UCG in the form of a chart to which they could easily make reference. Thus, if usage of the UCG is to be promoted in rural Uganda, there is need to explore context specific alternatives to making the UCG better accessible to the intended users [62, 64]. Different audiences have different needs, learning styles and preferences, therefore when coming up with alternatives, there is need to be sensitive to the different segments or subgroups and come up with interventions that will maximally appeal to the targeted audience [64].

### Barriers related to point-of-care access

The PCPs were faced with a point-of-care access challenge during the management of mental health problems; they did not have the opportunity to search for specific information at the point of the patient encounter when faced with a new condition because they did not have access to the UCG, thus reluctance to comply. In this study, point-of-care access is used to mean access to the UCG in whichever form including hard copy and softcopy.

Implementation studies show that the use of verbal prompts and visualization cues as engagement strategies promote uptake of the guidelines [65]. This is a potential strategy that could be utilized to improve integration of the mental health services into PHC in Uganda; verbal prompts such as regulatory measures to enforce adherence to the guidelines as well as visualization cues such as summarized guidelines either packaged in pocket size books or charts on the wall may go a long way in improving the point-of-care access.

Our study found that the rural health centers were good avenues for conducting research, and there were a number of donor funded projects with specific targets and various cues to help the PCPs attain the expected deliverables. This however was not the case for mental health; we found that mental health neither had a special program in the health centers nor specific targets to promote its screening and management at PHC level. As such, the PCPs felt that mental health was not among the priority areas because there was no support directed to it from anybody, thus directing their energies to where they are required to be accountable. Although it is good for health centers to be study sites, the limitation is that the health conditions that are not of interest to the funders tended to be neglected. The PCPs put more focus on health conditions that had set targets. In a study by Saraceno, van Ommeren [66], mental health investments in PHC are important but are unlikely to be sustained unless they are preceded by the development of community mental health services, to allow for training, supervision, and continuous support for primary care workers. In order to promote uptake of clinical guidelines, there is need for the policy makers to understand the local contexts of the health centers, and provide the relevant facilitation to allow for the local adaptation of guidelines [67, 68]. In addition, allowing for local consensus is important to change social norms and, improve guideline implementation [69].

### Barriers related to integrating trained mental health providers

Our study also found that PCPs trained as mental health providers and deployed at the various health centers were not entirely working in their area of training. They were not happy with the fact that they had to rotate to different wards providing general care as opposed to their area of interest (mental healthcare); this lowered their motivation to work. This however is a policy option that was adopted to address the issue of stigma [9].

When we analyzed the mental health integration policy, we found that: a) *integration of mental health services into PHC* can be likened to task-shifting mental health into general care so that all clinicians regardless of whether they received specialized training in providing mental healthcare or not assess for mental health problems [9]. This policy may in part compromise the quality of care provided if the PCPs are provided with specialized training in mental healthcare. This is in agreement with a study by Jerene, Biru [70], who note that task-shifting mental health to general medical care requires more than brief provider training to enable proper care. b) if *integration of mental health services into PHC* is to be effective, there is need to provide co-training of the general PCPs with the mental health providers, and actively promote local collaborations (internal consultation) [70] otherwise, the policy of mental health integration defeats the purpose of specialized training of PCPs in mental health (psychiatry).

In spite of the numerous barriers, we found that the PCPs who utilized the UCG found them useful and believed that this would improve the care provided; this is in line with a study that was looking at implementing clinical guidelines in psychiatry [67].

## Limitations

The results presented in our study are views from only clinical officers, nurses and midwives; we do not have the views of medial officers because they either did not consent to take part in the study or were not available at the time of the study. We therefore cannot rule out that they have divergent opinions.

Given that this was a case study of one district in one region of Uganda, we cannot confidently generalize the findings as applicable to other settings. There may be need for similar studies in the other regions of the country to confirm our findings.

## Conclusions

Efforts to achieve successful integration of mental health services into PHC need to as much as possible fit into the context of the implementers. Much as the UCG spell out step-by-step procedure on how to utilize the guidelines for the management of common disorders, that is not good enough. There is need for a) adapting the guidelines into local context, this would involve sensitizing the users about the content and making them as accessible as possible, b) have some form of cues to enforce implementation of the policy option, and c) see how best to optimize the available expertize (mental health providers) in the integration process so that the trained mental health providers do not feel deprived of the opportunity to practice in their area of interest.

The findings of this study are important because they are an eye opener to the fact that policy options or guidelines should not be generalized but rather context specific to the areas where they are to be implemented, thus promoting better uptake.

## Additional file

Additional file 1: Interview guide. (DOCX 17 kb)

## Abbreviations

COM-B: Capability opportunity and motivation to behavior
GUREC: Gulu University Research Ethics Committee
HC: Health center
HF: Health facility
PCP: Primary care provider
PHC: Primary health care
Sida: Swedish International Development Cooperation Agency
UCG: Uganda clinical guidelines
UMHCP: Uganda minimum health care package
UNCST: Uganda National Council for Science and Technology
WHO: World Health Organization
