# Supplementary material for: Barriers and facilitators to the integration of mental health services into primary healthcare: a qualitative study among Ugandan primary care providers using the COM-B framework
Source: BMC Health Serv Res. 2018 Nov 26;18:890. doi: 10.1186/s12913-018-3684-7 (PMC6258411; doi:10.1186/s12913-018-3684-7)
Supplement: Supplementary file 1 — Interview guide. (DOCX 17 kb) [file 12913_2018_3684_MOESM1_ESM.docx]

**Additional file 1: Overview of the Interview guide**

| **Domain** | **Constructs** | **Interview question** |
| --- | --- | --- |
| Capability | Psychological ability | What mental disorders do you commonly see at this facility?  Do you know about the UCG on management of common conditions?  If yes, have you looked through them?  What do you think the guidelines say about management of mental health problems?  Is screening for mental health problems something you usually do (Would you remember)?  What do you think about it as part of your role?  Would you remember to follow the guidelines when screening for mental health? How would that happen? |
|  | Physical ability | How do you screen them for mental health?  Have ever used the UCG? |
| Opportunity | Physical environment | To what extent does the surrounding environment facilitate or hinder screening for mental health problems?   - Space from where to screen the patients - Competing tasks or time constraints - Access to the UCG |
|  | Social environment | To what extent do social influences facilitate or hinder you from screening for mental health problems? (prompt for peers, seniors, patients, relatives)   - See others screening for mental health - See others using the UCG when screening - Support supervision when screening for mental health |
| Motivation | Reflective mechanism | What do you think about following the guidelines when screening for mental health?  Do you think you need to use the UCG to screen for mental health?  How easy or difficult do you think it is to follow the UCG when screening for mental health?  What challenges do you think are there in using the guidelines to screen and manage mental health problems?  What support would you need in order to use the UCG?  How confident are you that you can use the UCG to screen for mental health problems once they are provided?  How will you feel if do or do not follow the UCG to screen for mental health?  What would motivate you to screen for mental health?  What would motivate you to follow the UCG when screening for mental health? |
|  | Automatic mechanism | Does screening for mental health problems cause some emotions in you?   - If so, what? - To what extent do emotional factors facilitate or hinder you from screening for mental health problems?   What preparatory or introductory steps are there at health facility level to help you screen for mental health problems? Probes for   - Preparation on how to use the UCG - Measures (regulations) of encouraging you to screen for mental health |
